# Validation of the Spanish Version of the Work Group Emotional Intelligence Profile Short Version (WEIP-S) in the Sports Context

**DOI:** 10.3390/ijerph18020715

**Published:** 2021-01-15

**Authors:** Carlos Marchena-Giráldez, Jorge Acebes-Sánchez, Francisco J. Román, Miriam Granado-Peinado

**Affiliations:** 1 Faculty of Education and Psychology, Universidad Francisco de Vitoria (UFV), Pozuelo de Alarcón, 28223 Madrid, Spain; carlosalberto.marchena@ufv.es (C.M.-G.); miriam.granado@ufv.es (M.G.-P.); 2 Faculty of Health Sciences, Universidad Francisco de Vitoria (UFV), Pozuelo de Alarcón, 28223 Madrid, Spain; 3 Faculty of Psychology, Universidad Autónoma de Madrid (UAM), 28049 Madrid, Spain; f.javier.roman@uam.es

**Keywords:** emotional intelligence, sports, workgroup, validation, WEIP-S

## Abstract

Emotional intelligence (EI) is related to better performance in sports. To measure this construct, many tools have been developed and validated in the sports context. However, these tools are based on an individual’s ability to manage their own emotions, but do not consider the emotions of the rest of the team (teammates, coaches, etc.). In this regard, the Workgroup Emotional Intelligence Profile short version (WEIP-S) is a self-reported measure designed to measure the EI of individuals who are part of a team. The aim of this study was to validate the WEIP-S structure to measure EI in the sports context, and to analyze the psychometric properties of this tool in the sample in terms of validity and reliability. A cross-sectional study was conducted among 273 athletes to examine the reliability, factor structure, and evidence of validity (convergent, discriminant, nomological, and concurrent) of the WEIP-S. Confirmatory factor analysis showed that the original four-factor structure is the most appropriate for the sports context. Composite reliability was adequate for all factors except management of one’s own emotions, which also showed poor convergent validity. Evidence of convergent, discriminant, and nomological validity are discussed. This study represents an advance in the use of specific scales to measure EI in the sports context.

## 1. Introduction

Emotional intelligence (EI) has been a subject of great interest to researchers in different areas. Higher EI is related to mental, psychosomatic, and physical health outcomes [1]. In the educational context, higher EI is related to better academic performance and negatively related to aggressiveness [2,3]; for teachers, it is negatively related to burnout [4,5]. In the organizational context, different studies state that higher EI is related to higher scores in performance variables such as job satisfaction and team work effectiveness [6,7,8]. EI is also related to academic, professional, and career success [9,10,11]. Likewise, in the sports context, higher levels of EI are related to higher levels of physical activity [12,13].

EI can be defined as the ability to perceive accurately, appraise, and express emotions; the ability to generate feelings when they facilitate thought, understand the emotional knowledge, and regulate emotions to promote emotional and intellectual growth [14]. However, due to the interest and specialization of this variable in different contexts, new definitions have been created. Thus, emotions in the sports context are described as organized psychophysiological reactions to assess ongoing relationships with the environment [15,16,17]. EI has been of great interest in the sports context [18]. Different studies have stated that higher EI is related to better sports performance for athletes [18,19].

There is a wide scientific debate on which is the most appropriate tool to assess EI [20]. One of the most important reasons is the different approaches [21]. Petrides and Furnham [21] differentiated between trait EI and information-processing EI. Trait EI is related to the consistency of a specific behavior characterized by traits such as empathy, assertiveness, or optimism, so it will be integrated into the personality. This type of IE is evaluated through validated self-reported inventories such as the Trait-Meta Mood Scale (TMMS-24) [22], the Bar-On Emotional Quotient Inventory (EQ-i) [23], the Schutte Self Report Inventory (SSRI) [24], and the Trait Emotional Intelligence Questionnaire (TEIQue) [25]. On the other hand, information-processing EI refers to the ability to identify, express, and label emotions. This way of understanding IE is evaluated through measures of maximal (not typical) performance, such as the Emotional Intelligence Test (MSCEIT) by Mayer et al. [26]. These instruments have been designed and validated for the general population, without any specific reference to the sports field.

Despite the lack of validated instruments that measure EI in the sports context, the scientific literature shows that this is a relevant variable that can influence sports performance. Mills et al. [27] interviewed 10 expert coaches from the English Professional Soccer League and found that EI was considered important for successful progression, since players who know how to regulate their emotions and impulses adapt better to changing circumstances. In this sense, a lack of emotional competence could make it difficult to move up to a professional level. On the other hand, some research shows that a high level of EI in athletes can reduce their anxiety [28,29] and competition stress [30], and it is positively related to psychological skills such as self-talk, goal-setting, imagery, and relaxation skills [31].

Different studies have validated EI measurement instruments for the sports context [31,32], or have adapted the content to this field [33]. They used the SSRI, TMMS-24, and EQ-I as theoretical base models, which understand EI as an individual trait, but do not consider relationships with other people. In the organizational field, many studies have been conducted on team EI and team effectiveness or performance [34,35,36,37]. However, there is a lack of research on team EI in the sports setting [38], and this may be because there is no validated instrument that measures team EI in this context.

Workgroups are designed to bring together individuals for a common purpose, performance goals, and all members consider themselves responsible [39]. A unique tool for measuring workgroup EI is the Workgroup Emotional Intelligence Profile (WEIP) and its short version (WEIP-S) [40]. This questionnaire, designed and validated in the organizational context, focuses on abilities related to one’s own emotions and abilities related to the emotions of others. The final WEIP-S consists of four EI subscales related to (1) the awareness of one’s own emotions, (2) the management of one’s own emotions, (3) the awareness of others’ emotions, and (4) the management of others’ emotions. WEIP-S has been validated in the business context with French [34] and Spanish [41] employees in different workgroups. The Spanish version replicates the factor structure and has an adequate reliability rating, and the relations with other criteria and variables are similar to the original version [41]. In the same way, the French version of the WEIP-S has a four-factor structure and can measures through 16 items, and has good internal consistency and reliability [34]. However, in the sports context, the WEIP-S has only been validated in a Portuguese sample of football players, obtaining reliability and validity values similar to the original version in all dimensions except management of one’s own emotions, where the alpha value did not reach 0.70 [42]. Sports performance is measured by goals; these goals are not only for the athlete or team but for the coach, other athletes, psychologists, physiotherapists, etc. Team EI predicts team sports performance [43], however there is only one study that addresses EI as a workgroup variable in the sports context [42]. Thus, the present study aims to validate the Spanish version of the WEIP-S questionnaire for the measurement of EI in the sports context, and to analyze the psychometric properties of this tool in a sports sample in terms of validity and reliability.

## 2. Material and Methods

### 2.1. Participants

The sample in this study is made up of federated sportsmen and -women. Therefore, data collected by the Ministry of Culture and Sports were used to determine the sample size. In 2019, the percentage of federated athletes in Spain was 8.27% of the population [44]. Therefore, the minimum number of participants in our study was 117 (confident interval = 95%; margin of error = 5%; population portion = 8.27%). Here, we evaluated 273 athletes from different sport modalities. The sample was for convenience and non-random. Mean age was 24.33 years (*SD* = 8.98). Table 1 shows the specific characteristics of the participants. The inclusion criteria were as follows: practicing a sport in federated competitions (to ensure the competitive involvement of athletes in their sport), a minimum of one year practicing the sport, and regular sports practice (at least one hour a week). The exclusion criterion was having a diagnosis of a psychological disorder or being under psychological treatment.

### 2.2. Measures

First, participants filled out a sociodemographic questionnaire created ad hoc to collect information on different variables (age, sex, modality of sport, years and frequency of practice, etc.). They also completed the following standardized measures:

- Workgroup Emotional Intelligence short version (WEIP-S) [40] in the Spanish version by López-Zafra et al. [41]. The questionnaire comprised 16 items to assess EI in the workgroup context. Responses are given according to a Likert scale ranging from 1 (strongly disagree) to 7 (strongly agree) and are divided into four dimensions (with four items in each dimension): awareness of one’s own emotions (e.g., item 1: I can express my emotions to the members of my team); management of one’s own emotions (e.g., item 5: I respect the opinion of the members of my team, even if I think they are wrong); awareness of others’ emotions (e.g., item 9: I realize their true feelings even if they try to hide them); and management of others’ emotions (e.g., item 13: My enthusiasm can rub off on my team members). Internal consistency values ranged from good (α = 0.71) to excellent in all dimensions (α = 0.91).
- Trait Meta Mood Scale (TMMS-24) [22] in the Spanish version by Fernández-Berrocal et al. [45]. This self-reported measure is composed of 24 items to assess the individual’s EI. Responses are given according to a Likert scale ranging from 1 (never) to 5 (very often) and are divided into three dimensions (with eight items in each dimension): emotional attention, emotional clarity, and emotional repair. Spanish validation showed good values of internal consistency in all subscales (above α = 0.85), as well as temporal stability (from *r* = 0.60 to *r* = 0.83). This measure has also been validated in the sports context, showing adequate reliability and construct validity [46].
- Perceived Stress Scale (PSS) [47] in the Spanish version by Remor [48]. It consists of a one- dimensional scale composed of 14 items to assess perceived stress level in the last month (e.g., item 1: In the past month, how often have you been affected by something that happened unexpectedly?). Responses are given according to a Likert scale ranging from 0 (never) to 4 (very often). Psychometric property analysis showed good values of internal consistency (α = 0.81) and test-retest temporal stability (*r* = 0.73).
- Dysexecutive Questionnaire (DEX) [49], validated by Pedrero-Pérez et al. [50]. It consists of a questionnaire composed of 20 items to assess executive function. Responses are given according to a Likert scale ranging from 1 (never) to 5 (very often) and are divided into two dimensions (with 10 items in each dimension): disorganization-apathy (e.g., item 1: I have trouble understanding what others mean even if they say thing clearly) and disinhibition-impulsivity (e.g., item 2: I act without thinking, doing the first thing that comes to mind). Internal consistency of the Spanish version was found to be good (α = 0.87).
- Self-reported measures. We asked the participants about their perception of their sports performance (“In your opinion, your sport performance is …”) with four answer options (very good, good, medium, low), and their satisfaction with their performance (“I am satisfied with my sport performance”), with four answer options (totally agree, agree, disagree, totally disagree).

### 2.3. Procedure

Participation was requested by email to sports institutions in Spain, and by snowball sampling. The data recruitment was from 17 March to 15 July 2020. After reviewing general information about the purpose of the study, participants signed an informed consent form to express their agreement to participate in the study. In no case did participants receive any compensation; participation was entirely voluntary. Data collection was carried out by using the Google Forms platform. No personal data were required for the participants, to guarantee confidentially. The time of filling out the questionnaire was around 20 min. The study fully complied with the Helsinki Declaration and was approved by the Research Ethics Committee of the Universidad Francisco de Vitoria (16/2020).

### 2.4. Data Analysis

R software (https://www.r-project.org/) (R Core Team, Vienna, Austria) was employed to compute several analyses. First, descriptive statistics were computed for all psychological tests administered. Univariate and multivariate normality were tested with the Kolmogorov-Smirnov and Mardia tests, respectively.

**Figure 1:**
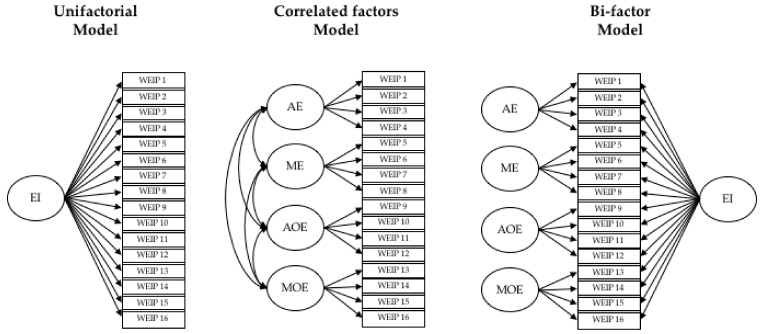
Models tested for Workgroup Emotional Intelligence Profile (WEIP-S). EI, emotional intelligence; AE, awareness of own emotions; ME, management of own emotions; AOE, awareness of others’ emotions; MOE, management of others’ emotions.

Secondly, the lavaan package was employed to compute several confirmatory factor analyses. Specifically, we compared three models: (1) unifactorial model; (2) correlated model with the four theoretical factors proposed by the authors [39]: awareness of own emotions (AE), management of own emotions (ME), awareness of others’ emotions (AOE), and management of others’ emotions (MOE); and (3) bi-factor model, where the correlation between the general EI factor and the specific factors are constrained to be zero (see Figure 1). The bi-factor model is advantageous for multidimensional constructs. Moreover, the advantages of the bi-factor model over the hierarchical model are well known [51]: (1) the general factor is easier to interpret; (2) general and specific influences on indicators can be examined simultaneously; (3) omega-hierarchical and omega-subscale can be computed to explore scale and subscale model reliability; and (4) the unique contribution of the specific and general factors can be assessed for predicting external criteria.

- Model fit was assessed using root mean square error of approximation (RMSEA) with a 90% confidence interval, comparative fit index (CFI), Tucker-Lewis index (TLI), and standardized root mean square residual (SRMR). It is considered that values > 0.95 for CFI and TLI and < 0.06 for RMSEA and SRMR indicate good model fit [52]. The fit of the models was compared using the chi-square difference test. In addition, Akaike information criterion (AIC) [53], Bayesian information criterion (BIC) [54], and sample-size-adjusted BIC [55] were also reported to compare the fit of the models. Lower values indicate a better fit [56].
- The convergent and discriminant validity of the CFA model was also explored. Convergent validity is related to whether a latent variable is well estimated with the selected items. The factor loading of the indicators and the average variance extracted (AVE) must be considered for convergent validity. The AVE value should be equal to or higher than 0.50. Discriminant validity measures the degree of difference between overlapping factors [57]. Discriminant validity was assessed using cross-loading of indicators with the Fornell and Larcker criterion [58] by comparing the squared correlations between latent variables against their AVE scores. AVE should be larger than the squared correlation with any other construct. Moreover, composite reliability (CR) [59] was computed to estimate the internal consistency, with a threshold of 0.70 indicating sufficient internal consistency reliability.

Next, nomological validity was explored computing the correlation coefficients between WEIP-S scales and additional measures. Trait-Meta Mood Scale (TMMS), Dysexecutive Questionnaire (DEX), and Perceived Stress Scale (PSS) were computed to explore the association of WEIP-S with other related constructs. Higher correlations are expected between WEIP-S and TMMS-24 scales and PSS, while lower correlations are expected between WEIP-S and DEX.

Finally, multiple regression models were computed to predict two self-reported criteria (sport performance perception and satisfaction with performance) using the WEIP-S subscales, TMMS-24 scale, PSS, and DEX. The WEIP-S subscale scores were saved from the CFA model using the predict function of R software. The lm function of R software was employed to estimate the regression models.

## 3. Results

### 3.1. Descriptive Analysis

Table 2 shows the main descriptive analysis of the measured variables. Univariate normality was explored using the Kolmogorov-Smirnov test. Univariate normality was not assumed (*p* < 0.05) for the WEIP-S data, disorganization/apathy subscale of DEX, and self-reports on sport performance perception and satisfaction with performance. In addition, Mardia’s test was computed to assess multivariate normality. Multivariate normality was not assumed for WEIP-S (skewness = 5.7, *p* < 0.001; kurtosis = 313.67, *p* < 0.001). Therefore, nonparametric tests were applied.

### 3.2. Confirmatory Factor Analysis, Convergent and Discriminant Validity

- Three models were tested for the WEIP-S: the unifactorial model, four correlated factors (original model), and the bi-factorial model (see Figure 1). Maximum likelihood method (MLM) estimation [60] was used given the non-normal distribution of the data. Robust fit indices for each model tested are presented in Table 3 [61].
- The fit indices for the unifactorial model were unacceptable. The correlated and bi-factor models obtained similar fit indices with RMSEA values around 0.06, CFI higher than 0.95, and SRMR lower than 0.05. The model comparison also showed a similar fit for correlated and bi-factor models (*p* > 0.05). The AIC, BIC, and ADJ BIC values were lower for the correlated model. In addition, some factor loadings in the bi-factor model were non-significant and lower than 0.30. Therefore, we considered the correlated model as the best way to summarize the WEIP-S data.

Table 4 shows the specific data for the correlated model. Correlations between factors were moderate (range: 0.431–0.583). Convergent validity was assessed by exploring the factor loadings and average variance extracted (AVE). The factor loadings of each factor were statistically significant (*p* < 0.001); AVE was higher than 0.50 except for the ME factor. Therefore, the only construct with unsatisfactory convergent validity was the ME factor. In terms of discriminant validity, the AVE of the two factors was greater than the square of the correlation between the factors in all comparisons. Therefore, we can assume discriminant validity between the subscales of the WEIP-S.

Finally, the CR values were 0.84 for the AE factor (awareness of own emotions), 0.65 for the ME factor (management of own emotions), 0.83 for AOE (awareness of others’ emotions), and 0.90 for the MOE factor (management of others’ emotions).

In addition, the A web platform (http://www.quantpsy.org/rmsea/rmsea.htm [62]) was employed to calculate the power level achieved with our data in the correlated CFA model. The outcome showed a power level of 0.99. The data included in the estimation were degrees of freedom = 98; sample size = 273; α = 0.05; null RMSEA = 0.05; alternative RMSEA = 0.1.

### 3.3. Nomological Validity

Spearman correlation was computed between WEIP-S, TMMS, PSS, and DEX to explore the associations between related measures. Specifically, we expected higher associations between WEIP-S and TMMS and PSS and lower associations between WEIP-S and DEX. Table 5 shows the Spearman correlations between variables.

Correlations of WEIP-S subscales with TMMS-24 subscales show a significant positive correlation between subscales related to others’ emotions (awareness and management) with all TMMS-24 subscales. However, for subscales related to one’s own emotions (awareness and management), statistical significance was found with the emotional clarity and emotional repair subscale of the TMMS-24. The Perceived Stress Scale was inversely related to the scales related to management of emotions (one’s own and others’ emotions) of the WEIP-S. Finally, all WEIP-S subscales showed a significant inverse correlation with the disorganization/apathy subscale and DEX total score, except for awareness of others’ emotions. For the disinhibition/impulsivity subscale, awareness and management of own emotions were inversely correlated.

### 3.4. Multiple Regression Models

Two self-reported measures (sport performance perception and satisfaction with performance) were predicted using the psychological measures as predictors (WEIP-S subscales, TMMS-24 subscales, PSS, and DEX subscales). These two measures sports performance were only predicted by the management of others’ emotions subscale of WEIP-S (*β* = 0.307; *p* < 0.001 for sport performance and *β* = 0.200; *p* < 0.05). The explained variance of the criteria was 13.04% for sports performance and 12.42% for satisfaction with performance. See Table 6 for details.

## 4. Discussion

The present study aimed to validate the factor structure of the WEIP-S for athletes and to analyze the psychometric properties. These objectives were motivated by the lack of instruments to measure EI in the sport context. Regarding the WEIP-S structure, the CFA results showed that both the default model with correlated factors and the bi-factor model were adequate for the collected sample. The results of reliability and convergent validity were good in three of four factors, except for ME. Values of discriminant validity between WEIP-S factors were appropriate, which seems to indicate a correct theoretical delimitation among the factors.

Other validation studies found a similar structure of the WEIP-S in both the job context [34,40,41] and the sports context, such as the Portuguese version of the WEIP-S that was validated in a sample of 150 soccer players [42]. The results of previous validations are also similar in relation to the reliability of the ME factor, showing the poorest value of internal consistency. Specifically, in the case of the Portuguese version validated in the sports context, the value of internal consistency was 0.62 for the ME factor, near our value of reliability. Despite some authors, such as DeVellis [63], suggesting that 0.60 can be used as the cut-off point in social sciences, the value of convergent validity of ME leads us to consider some problems in the measure of this factor. When analyzing the content of the items belonging to this factor, only one of the four items refers directly to one’s own emotions (item 6: When I am frustrated with fellow team members, I can overcome my frustration). The rest of the items are more related to managing conflicts with team members, which requires behavioral skills, but not necessarily emotional management. For example, in item 5 (I respect the opinions of the members of my team, even if I think they are wrong), it is assumed that different opinions necessarily provoke emotions that must be managed. However, athletes may answer this item considering only what they would do in this situation, not what they would feel.

In the correlation between measures to study nomological validity, we found correlations between all WEIP-S factors and the TMMS-24 subscales, except for the AE and ME factors and emotional attention of TMMS-24. This finding is different from the result obtained in the study of the Portuguese version, which found correlations between all subscales of both questionnaires [42]. However, in the study carried out by López-Zafra et al. [41], no correlation was found between emotional attention of the TMMS-24 and ME and MOE factors. These findings may highlight some difficulties in the use of an emotional attention measure as a linear variable. Following the TMMS-24, individuals who score high on emotional attention pay too much attention to their emotions, which could lead to higher levels of anxiety and make it difficult to manage emotions [44].

Regarding the perceived stress measure, only the two factors of managing emotions (ME and MOE factors) showed significant correlations. This finding seems congruent if we analyze the content of most of the items of the PSS that refer to coping with stressful situations in the last month. Thus, managing stress necessarily requires managing emotions [64]. The rest of the items of the PSS refer to feelings resulting from stressful situations experienced in the last month. However, this perception does not to have to be related to the remaining factors of the WEIP-S, which refers to the ability to express emotions to team members (AE factor) and to identify emotions of team members (AOE factor). In simple terms, athletes can perceive the emotions resulting from experiencing a stressful situation in the last month, but may not be able to express it to team members or recognize emotions in others. The correlation of the management of emotions with perceived stress is related to other studies revealing the relationship between EI and a protective role against stress [18].

The correlations between WEIP-S factors and the DEX were mostly significant but in the negative direction. In fact, this is congruent if we consider that people with dysexecutive syndrome show difficulties in emotion regulation, from emotional impulsivity to apathy or disorganized emotional patterns [65]. However, no correlation was found between the disinhibition/impulsivity subscale of the DEX and the factors of the WEIP-S related to the others’ emotions (AOE and MOE), which is congruent because no item of the DEX refers to the emotions of others.

Finally, the predictive potential of the MOE factor of sport performance and satisfaction with performance must be noted. This idea suggests that how athletes cope with the emotions of their teammates (in terms of enthusiasm and vitality, as the items indicate) is important to favor performance. In fact, some studies showed that being able to interact and handle conflicts with team members is related to performance [66]. This shows the importance of including relationship skills (conflict management, adequate interactions, etc.) in performance improvement programs [67].

Our work has some limitations to be addressed in future studies. First, the sample analyzed was heterogeneous in terms of sex distribution and type of sport practiced. This limitation makes it necessary to take the results about the factorial structure of the WEIP-S in the sports context with caution. Thus, it requires an invariance analysis to compare genders and different sports. Second, the measurement of sport performance was based on two self-reported questions measured concurrently with the EI measure. Thus, considering that performance is a complex phenomenon related to psychological, physical, tactical, technical, and theoretical factors [68], this measure seems to be insufficient. Therefore, it would be advisable to use different measures to assess sports performance using standardized questionnaires, subjective evaluation of performance by athletes and coaches, and sports achievement. Furthermore, to guarantee predictive value, it would be advisable to take those measures at the end of the sports season. Finally, related to the latter, our study is cross-sectional, so any possible causality between EI and the rest of the variables analyzed must be viewed with caution.

Despite these limitations, our study has some implications for sports practice. It emphasizes the importance of the rest of the team in measuring the EI of athletes, an element that is generally forgotten when measuring this construct. This suggests that, so far, measuring EI has been centered solely on the individual, therefore the entire construct was not being measured. Following this idea, programs aiming to improve sports performance through EI are probably not considering all aspects of the construct, which could limit the effectiveness of interventions. Thus, future programs designed to manage stress and improve performance by enhancing EI in sports should take into account these aspects. For that purpose, measuring EI at both baseline and post-treatment is essential, and the WEIP-S seems to be a suitable tool for measuring all aspects of the construct.

## 5. Conclusions

The results of the present study suggest that the WEIP-S questionnaire represents a good proposal to measure EI in the sports context, considering the importance of both one’s own emotions and the emotions of others (teammates, technical teams). The four-factor structure is the most appropriate model to measure EI in a sample of athletes. The results of reliability and evidence of convergent and discriminant validity reveal that the WEIP-S is a promising instrument to measure EI in the sports context. Moreover, the MOE factor appears to be a predictor of perceived performance and satisfaction with performance. However, some limitations should be considered for future studies, such as some problems with the ME factor in terms of reliability and convergent validity.

## Figures and Tables

**Table 1:** Characteristic of the study sample.

Variables	N	%
Sex		
Male	112	41%
Female	161	59%
Worker		
Yes	141	51.6%
No	132	48.4%
Sport		
Football	91	33.3%
Handball	53	19.4%
Indoor football	17	6.2%
Athletics	15	5.5%
Triathlon	14	5.1%
Basketball	13	4.8%
Volleyball	11	4%
Other	59	21.26%
Competition level		
Local	16	5.9%
Provincial	91	33.3%
National	144	52.7%
International	22	8.1%
Years of practice		
Up to 1 year	2	0.7%
1 to 5 years	40	14.7%
5 to 10 years	79	28.9%
Up to 10 years	152	55.7%
Weekly hours of training		
Up to 2 h	7	2.6%
2 to 7 h	125	45.8%
7 to 14 h	101	37%
Up to 14 h	37	13.6%

**Table 2:** Descriptive statistics for psychological tests and self-reported performance.

	*M*	*SD*	Skewness (z-Score)	Kurtosis (z-Score)
WEIP-S: AE	2.78	4.99	–6.05	2.62
WEIP-S: ME	22.79	3.69	–7.69	9.63
WEIP-S: AOE	2.37	4.23	–5.22	3.37
WEIP-S: MOE	22.72	4.28	–9.87	1.00
TMMS: EA	28.60	6.03	–2.03	–1.71
TMMS: EC	29.28	6.03	–3.27	0.86
TMMS: ER	3.32	5.80	–3.07	–0.51
PSS	24.72	8.02	–0.76	–2.03
DEX: DA	23.19	7.06	2.85	–1.68
DEX: DI	25.32	6.23	1.44	–0.35
DEX level	48.51	12.24	1.85	–0.93
Sport performance perception	2.86	0.69	–2.90	1.45
Satisfaction with performance	2.89	0.64	–2.32	1.85

WEIP-S, Workgroup Emotional Intelligence Profile short version; TMMS, Trait-Meta Mood Scale; PSS, Perceived Stress Scale; DEX, Dysexecutive Questionnaire; AE, awareness of own emotions; ME, management of own emotions; AOE, awareness of others’ emotions; MOE, management of others’ emotions; EA, emotional attention; EC, emotional clarity; ER, emotional repair; DA, disorganization/apathy; DI, disinhibition/impulsivity.

**Table 3:** Fit index values for WEIP-S models.

	Model Descriptive Statistics		
Fit Measure	Unifactorial	Correlated Factor	Bi-Factor
χ^2^	58.87	166.39	158.09
	(*df* = 104); *p* < 0.001	(*df* = 98); *p* < 0.001	(*df* = 88); *p* < 0.001
RMSEA	0.153	0.058	0.062
[CI; 90%]	[0.141–0.166]	[0.043–0.073]	[0.046–0.077]
CFI	0.649	0.952	0.951
TLI	0.591	0.941	0.933
SRMR	0.109	0.048	0.044
AIC	13,666.345	13,085.892	13,093.295
BIC	13,781.848	13,223.052	13,266.549
ADJ BIC	1368.384	13,102.564	13,114.353
	**Model Comparison (dif. χ^2^; *p*** **-Value)**		
Unifactorial		224.11 (*df* = 6);	322.84 (*df* = 16);
		*p* < 0.001	*p* < 0.001
Correlated			8.9193 (*df* = 10);
			*p* = 0.540

RMSEA, Root Mean Square Error of Approximation; CI, Confidence Interval; CFI, Comparative Fit Index; TLI, The Tucker-Lewis Index; SRMR, Standardized Root Mean Square Residual; AIC, Akaike’s Information Criterion; BIC, Bayesian Information Criterion; ADJ BIC, Adjusted Bayesian Information Criterion.

**Table 4:** Factor structure for WEIP-S model.

	Factor Loadings				R^2^
	AE	ME	AOE	MOE	
WEIP 1	0.877				0.768
WEIP 2	0.808				0.653
WEIP 3	0.679				0.461
WEIP 4	0.649				0.421
WEIP 5		0.584			0.341
WEIP 6		0.586			0.343
WEIP 7		0.52			0.271
WEIP 8		0.56			0.314
WEIP 9			0.72		0.518
WEIP 10			0.815		0.664
WEIP 11			0.801		0.642
WEIP 12			0.62		0.384
WEIP 13				0.878	0.771
WEIP 14				0.794	0.631
WEIP 15				0.824	0.679
WEIP 16				0.827	0.685
**Average Variance Extracted (AVE) and Shared Variance Estimates**					
AE	**0.576**	0.513	0.507	0.557	
ME	0.263	**0.317**	0.458	0.431	
AOE	0.257	0.210	**0.552**	0.583	
MOE	0.310	0.186	0.340	**0.691**	

WEIP, Workgroup Emotional Intelligence Profile; AE, awareness of own emotions; ME, management of own emotions; AOE, awareness of others’ emotions; MOE, management of others’ emotions. Note: Correlations are above the diagonal, squared correlations are below the diagonal, and AVE estimates are on the diagonal. Bold highlights convergent validity values. Bold: convergent validity values.

**Table 5:** Spearman correlation among variables.

	ME	AOE	MOE	TMMS: EA	TMMS: EC	TMMS: ER	PSS	DEX: DA	DEX: DI	DEX Level
AE	0.55 **	0.53 **	0.55 **	0.05	0.19 **	0.21 **	−0.12	−0.23 **	−0.10 **	−0.19 **
ME		0.59 **	0.49 **	−0.02	0.21 **	0.34 **	−0.20 **	−0.29 **	−0.22 **	−0.29 **
AOE			0.66 **	0.17 **	0.22 **	0.29 **	−0.09	−0.24 **	−0.04	−0.16 **
MOE				0.14 *	0.26 **	0.38 **	−0.17 **	−0.26 **	−0.07	−0.19 **

* *p* < 0.05; ** *p* < 0.01. TMMS, Trait-Meta Mood Scale; PSS, Perceived Stress Scale; DEX, Dysexecutive Questionnaire; AE, awareness of own emotions; ME, management of own emotions; AOE, awareness of others’ emotions; MOE, management of others’ emotions; EA, emotional attention; EC, emotional clarity; ER, emotional repair; DA, disorganization/apathy; DI, disinhibition/impulsivity.

**Table 6:** Regression outcomes.

	**Sport Performance**				
	**Estimate**	**Std. Error**	**t-Value**	**Standardized β**	***p*-Value**
**(Intercept)**	**3.149**	**0.422**	**7.469**		**<0.001**
AE	0.058	0.049	1.183	0.099	0.238
MO	–0.079	0.099	–0.793	–0.066	0.429
AOE	–0.015	0.070	–0.220	–0.019	0.826
**MOE**	**0.202**	**0.056**	**3.574**	**0.307**	**<0.001**
TMMS: EA	0.006	0.007	0.784	0.050	0.434
TMMS: EC	–0.010	0.008	–1.301	–0.088	0.194
TMMS: ER	0.002	0.008	0.243	0.017	0.808
PSS	–0.004	0.006	–0.753	–0.052	0.452
DEX: DA	–0.006	0.009	–0.615	–0.057	0.539
DEX: DI	0.001	0.009	0.092	0.008	0.927
	**Satisfaction with Performance**				
	**Estimate**	**Std. Error**	**t-Value**	**Standardized β**	***p*-Value**
**(Intercept)**	**2.940**	**0.391**	**7.519**		**<0.001**
AE	0.080	0.045	1.775	0.149	0.077
MO	–0.012	0.092	–0.126	–0.010	0.8998
AOE	–0.063	0.065	–0.961	–0.082	0.3372
**MOE**	**0.122**	**0.052**	**2.328**	**0.201**	**0.0207**
TMMS: EA	–0.001	0.007	–0.206	–0.013	0.8366
TMMS: EC	–0.008	0.007	–1.089	–0.074	0.2772
TMMS: ER	0.014	0.008	1.798	0.125	0.0734
PSS	–0.011	0.006	–1.923	–0.134	0.0555
DEX: DA	0.003	0.008	0.366	0.034	0.7147
DEX: DI	0.000	0.009	–0.015	–0.001	0.9877

WEIP-S, Workgroup Emotional Intelligence Profile short version; TMMS, Trait-Meta Mood Scale; PSS, Perceived Stress Scale; DEX, Dysexecutive Questionnaire; AE, awareness of own emotions; ME, management of own emotions; AOE, awareness of others’ emotions; MOE, management of others’ emotions; EA, emotional attention; EC, emotional clarity; ER, emotional repair; DA, disorganization/apathy; DI, disinhibition/impulsivity. Bold data indicate significant variables.

## Data Availability

The data presented in this study are available on request from the coressponding author. The data are not publicly available due to restrictions of privacy.
